# Novel Insights in the Rehabilitation of Neglect

**DOI:** 10.3389/fnhum.2013.00780

**Published:** 2013-11-15

**Authors:** Luciano Fasotti, Marlies van Kessel

**Affiliations:** ^1^Rehabilitation Medical Centre Groot Klimmendaal, SIZA Support and Rehabilitation, Arnhem, Netherlands; ^2^Donders Institute for Brain, Cognition and Behaviour, Radboud University Nijmegen, Nijmegen, Netherlands; ^3^Medisch Spectrum Twente Hospital Group, Enschede, Netherlands

**Keywords:** visuospatial neglect, treatment outcome, stroke, rehabilitation, novel treatments

## Abstract

Visuospatial neglect due to right hemisphere damage, usually a stroke, is a major cause of disability, impairing the ability to perform a whole range of everyday life activities. Conventional and long-established methods for the rehabilitation of neglect like visual scanning training, optokinetic stimulation, or limb activation training have produced positive results, with varying degrees of generalization to (un)trained tasks, lasting from several minutes up to various months after training. Nevertheless, some promising novel approaches to the remediation of left visuospatial neglect have emerged in the last decade. These new therapy methods can be broadly classified into four categories. First, non-invasive brain stimulation techniques by means of transcranial magnetic stimulation (TMS) or transcranial direct current stimulation (tDCS), after a period of mainly diagnostic utilization, are increasingly applied as neurorehabilitative tools. Second, two classes of drugs, dopaminergic and noradrenergic, have been investigated for their potential effectiveness in rehabilitating neglect. Third, prism adaptation treatment has been shown to improve several neglect symptoms consistently, sometimes during longer periods of time. Finally, virtual reality technologies hold new opportunities for the development of effective training techniques for neglect. They provide realistic, rich, and highly controllable training environments. In this paper the degree of effectiveness and the evidence gathered to support the therapeutic claims of these new approaches is reviewed and discussed. The conclusion is that for all these approaches there still is insufficient unbiased evidence to support their effectiveness. Further neglect rehabilitation research should focus on the maintenance of therapy results over time, on a more functional evaluation of treatment effects, on the design and execution of true replication studies and on the exploration of optimal combinations of treatments.

## Introduction

Visuospatial neglect is defined as an impairment whereby patients do not attend to visual stimuli or do not explore the visual half-space contralateral to their cerebral lesion (Heilman et al., 1993). It is usually the consequence of damage to the right hemisphere, most often due to an ischemic stroke. Visuospatial neglect is a major cause of disability, impairing the ability to perform a large range of everyday activities. Not eating food on the left part of the dish, bumping into obstacles on the left side, reading incomplete sentences in newspapers and ignoring objects on the left side are only a few impairments putting at risk the independence of stroke patients with left visuospatial neglect. Even without obvious signs of visuospatial neglect, stroke patients may suffer from subtle signs of neglect under increased attentional load (Bonato et al., 2010, 2013; Van Kessel et al., 2013a). Moreover, visuospatial neglect is often associated with other disabling symptoms like anosognosia and somatoparaphrenia. These co-morbidities may hamper the treatment of visuospatial neglect (see for example Borghese et al., 2013). Although some spontaneous recovery might take place until 2 or 3 months after stroke, visuospatial neglect persists in about one third of the patients (Kerkhoff and Schenk, 2012), leading to a chronic condition. More precisely, by using intensive serial measurements in the first months after stroke, Nijboer et al. (2013) were able to follow the exact course of recovery of visuospatial neglect in a group of 51 patients, using a line bisection and a letter cancelation test. The results show that after 12–14 weeks the recovery curves, as measured by a reduction of errors, grow flat, and spontaneous neurological recovery from neglect becomes invariant. Visuospatial neglect not only impairs patients in various visuospatial tasks, it is also associated with other consequences of stroke like problems with postural control, standing balance, and walking (Pérennou, 2006; Van Nes et al., 2009). It is considered to be a crucial factor influencing rehabilitation outcome, often leading to poor recovery from stroke (Jehkonen et al., 2006; DiMonaco et al., 2011; Vossel et al., 2013).

Given these premises, it is obvious that visuospatial neglect has been a target for rehabilitation since a long time. Starting in the early 1970s many rehabilitation techniques have been proposed to alleviate and reduce the problems generated by left visuospatial neglect. In a recent review Luauté et al. (2006a) distinguish and describe18 different approaches to the rehabilitation of neglect. In the present review we will describe the studies characterizing four of these approaches that have emerged since approximately a decade: prism adaptation (PA), virtual reality (VR) training, non-invasive brain stimulation (NIBS), and pharmacological therapies. Table S1 in Supplementary Material gives an overview of these studies (McIntosh et al., 2002, Angeli et al., 2004, Dijkerman et al., 2004, Jacquin-Courtois et al., 2008, Nijboer et al., 2011, Bauer et al., 2012, Luauté et al., 2012).

## Non-Invasive Brain Stimulation

The use of NIBS to improve impaired cognitive processes in neurologically impaired patients has recently received much attention (e.g., Miniussi and Vallar, 2011). More specifically, in neglect research, Transcranial Magnetic Stimulation (TMS) and transcranial Direct Current Stimulation (tDCS) have been used to ameliorate the symptomatology of patients with visuospatial disorders. With the aim to improve the duration of the after-effects of non-invasive stimulation methods, a particular form of TMS called Theta Burst Stimulation (TBS) has lately been introduced.

In order to understand the different forms of modulation of visuospatial functions by NIBS it is useful to describe the networks of attention involved in visuospatial neglect and to clarify the concept of interhemispheric rivalry. Visuospatial neglect is more and more seen as originating from a disruption of fronto-parietal networks of attention, particularly those of the right hemisphere (Thiebaut de Schotten et al., 2005; Bartolomeo et al., 2007; Committeri et al., 2007). Moreover, as proposed by Kinsbourne (1977, 1994), both parietal cortices also exert reciprocal interhemispheric inhibition. Therefore, injuries to the parietal areas of the right hemisphere do not only depress the activity of this area, they also cause disinhibition of the homolog areas of the left hemisphere. This overactivation of the left hemisphere aggravates the tendency of patients with visuospatial neglect to attend to the right and to neglect the left side. Empirical evidence for interhemispheric rivalry stems from the observation of patients with visuospatial neglect and from imaging research. Vuilleumier et al. (1996) observed a patient who had sequential strokes in both hemispheres. A first right-sided parieto-occipital infarct resulted in a severe left-sided neglect. However, about a week later, after a second infarct located in the left frontal lobe, the neglect symptoms abruptly subsided. In an fMRI study, Corbetta et al. (2005) noticed that in patients with visuospatial neglect, the intact left hemispheric orienting mechanism was relatively hyperactive. Recovery from neglect after 39 weeks showed a strong reactivation in several right hemisphere but also many left hemisphere regions, with a reduction of the activation imbalance between both hemispheres.

Starting from the idea of interhemispheric rivalry in visuospatial neglect, three non-invasive stimulation methods are basically conceivable: stimulation of the damaged right hemisphere brain areas, inhibition of the hyperactive intact left hemisphere, or both. Till now, the majority of NIBS studies targeting visuospatial neglect has been aimed at the inhibition of the left hemisphere.

Oliveri et al. (2001) were the first to apply contralesional parietal rTMS to five patients with right brain damage and two patients with left brain damage, all suffering from contralateral visuospatial neglect. rTMS was given during the presentation of bisected lines. Each transcranial stimulus train consisted of 10 stimuli delivered at a repetition frequency of 25 Hz during 400 ms. These trains started simultaneously with the appearance of the bisected lines on a monitor screen. After presentation, the subjects had to make a forced-decision about the length of the two bisected segments of each line with three response possibilities: equal, longer right, or longer left. To control for unspecific effects of rTMS, sham magnetic stimulation was intermingled with “real” rTMS trains. The results showed that rTMS of the unaffected hemisphere transiently decreased the magnitude of visuospatial neglect in both right and left lesioned patients as represented by wrong judgments, when compared with baseline (without rTMS) and sham rTMS trials.

Two years later, Brighina et al. (2003) applied low-frequency 1 Hz rTMS trains of 900 pulses in seven sessions over 14 days to three neglect patients with right brain damage. The pulses were given over the contralesional left parietal cortex. Visuospatial performance was assessed with the same task as in the Oliveri et al. (2001) study, namely making length judgments of prebisected lines presented on a computer screen. Unlike the Oliveri study, in which these judgments had to be given online, in the Brighina et al., study, the visuospatial line judgment task was administered four times: 15 days before treatment (T1), at the beginning of the treatment (T2), at the last day of the treatment (T3), and 15 days after (T4). At T1 and T2, a strong rightward bias was present in the patients. A significant amelioration of this bias was found after training (T3) and this improvement was still present 15 days after the end of the treatment (T4).

Other studies with small right brain lesioned patient groups and no control condition, using low-frequency rTMS inhibiting the left parietal cortex are those of Shindo et al. (2006), Koch et al. (2008), Song et al. (2009), and Lim et al. (2010). In the Shindo et al. (2006) study, six sessions of rTMS improved the performance of two right brain-damaged patients on several subtests of the Behavioral Inattention Test (BIT) up to 6 weeks after treatment. After a single low-frequency rTMS session, Koch et al. (2008) observed an improvement in the naming of visual chimeric figures in 12 right brain-damaged patients and in the Song et al. (2009) trial, two sessions of rTMS per day during 14 days ameliorated line bisection and line cancelation for up to 14 days after treatment in 7 patients with right brain damage. Lim et al. (2010) gave 1 Hz trains of 900 pulses for 5 days per week during 2 weeks to seven patients with right brain damage. They found that after training, line bisection had significantly improved, whereas line cancelation did not show gains. This dissociation can be explained by assuming that different brain areas underlie these tasks (see Ellison et al., 2004).

In contrast, one of the rare investigations in which the damaged right hemisphere was directly stimulated comes from Ko et al. (2008). Fifteen subacute stroke patients with visuospatial neglect after right hemisphere damage were recruited for this study. The study was designed as a double-blind, cross-over, sham-controlled experiment. All of the patients were stimulated with anodal (positive stimulation) and with sham tDCS in a counterbalanced and randomized order, with a 48-h interval between the two tDCS sessions. Anodal tDCS applied to the right posterior parietal cortex resulted in significant improvements of performance in a figure cancelation and a line bisection task immediately after brain polarization.

Sparing et al. (2009) tested the idea of interhemispheric rivalry most exhaustively. They treated 10 patients suffering from left visuospatial neglect with tDCS under the following conditions: (1) Anodal tDCS of the intact posterior parietal left hemisphere, (2) Cathodal (inhibiting) tDCS of the same area, (3) Anodal tDCS of the lesioned posterior parietal right hemisphere, and (4) Sham tDCS of the same hemisphere. The tDCS sessions were carried out on two separate days, with an intersession interval of at least 3 h and in a counterbalanced order of conditions across subjects. The authors conclude that both the inhibitory effect of cathodal tDCS applied over the intact left hemisphere as well as the facilitatory effect of anodal tDCS over the lesioned right hemisphere reduce symptoms of visuospatial neglect in a line bisection task but not on the neglect subtest of the TAP (Zimmermann and Fimm, 1995). Both tasks were administered before, immediately after and 20 min after the respective tDCS conditions.

Although the effects of rTMS seem to outlast the mere stimulation period, as shown above, these effects are only transient and their therapeutic benefits seem limited. In animal research, long-term potentiation (LTP) and long-term depression (LTD) of synaptic strength have been obtained with TBS. TBS is a high-frequency stimulation that is spaced at a frequency that mimics the theta wave, a spontaneous 5–7 Hz neural rhythm (Abraham, 2003).

As a proof-of-principle, Nyffeler et al. (2009) showed that several trains of TBS given to the left posterior parietal cortex of 11 neglect patient increased the number of perceived left visual targets for up to 32 h. Recently Koch et al. (2012) have investigated the efficacy of continuous TBS in 10 sessions over 2 weeks. The TBS trains were again applied to the left posterior parietal cortex of 18 neglect patients in the subacute stage of their illness. Scores on the BIT improved by 16.3% immediately after TBS application and by 22.6% at 1 month follow-up. In a double-blind, sham-controlled experiment, Cazzoli et al. (2012) applied four TBS trains to the left posterior parietal cortex of 16 neglect patients over two consecutive days. This resulted in a 37% improvement in the spontaneous everyday neglect behavior of the patients as measured by the Catherine Bergego Scale. This improvement was still present at 3 weeks after stimulation. The amelioration in neglect behavior was accompanied by better performances on several neglect tests. A control group of eight no-treatment (sham-stimulation) neglect patients did not show any progress.

## Pharmacological Therapies

According to Singh-Curry and Husain (2010), two classes of drugs have been investigated for their potential therapeutical effects in the rehabilitation of neglect: dopaminergic and noradrenergic drugs. Dopamine and noradrenaline play essential roles in attention and thinking. They contribute to maintaining alertness, increasing focus and sustaining thought, and cognitive effort. A majority of trials have studied dopaminergic drugs, whereas noradrenergic compounds have only rarely been investigated.

The modulation of dopaminergic activity through pharmacological agents has produced mixed results in older as well as in more recent studies. Recent studies include the use of levodopa (Mukand et al., 2001) and amantadine (Buxbaum et al., 2007). Significant improvements were found on selected subtests of the BIT (conventional as well as behavioral subtests) and on the Functional Independence Measure (FIM, Keith et al., 1987) in three out of four neglect patients, after 1 week of treatment with carbidopa l-DOPA (Mukand et al., 2001). A small trial with amantadine administered to four neglect patients (Buxbaum et al., 2007) was performed using a double-blind, placebo-controlled design. Care was taken to obtain a stable baseline of performance in the first placebo phase, in order to make sure that changes in the amantadine administration stage were not due to random variation. Also, neglect was tested thoroughly with a large array of tests, a naturalistic action test (NAT, Schwartz et al., 2002) and the FIM. The results showed that a vast majority of the 17 measures employed showed no improvement. The most recent study (Gorgoraptis et al., 2012) investigated the effects of the dopamine agonist rotigotine on visuospatial neglect. The study was set-up as a double-blind, randomized, placebo-controlled ABA investigation with three phases: baseline, rotigotine administration, and return to baseline. The duration of each phase was randomized within limits and 16 neglect patients were included. Outcome measures were visual neglect tasks, visual working memory tests, selective attention and sustained attention tasks, and a measure of motor control. The results showed an improvement in visual search while on rotigotine, with the number of targets found on the left increasing by 12.8% and a spatial bias reduced by 8.1%, in comparison with being off rotigotine. Improvement in visual spatial search was associated with an amelioration of selective attention, but not with alterations in working memory, sustained attention, or motor performance.

Only one trial with noradrenergic medication has recently been performed. Malhotra et al. (2006) carried out a proof-of-principle trial with guanfacine, a noradrenergic agonist. Three chronic neglect patients participated in a double-blind cross-over trial and were tested six times with an extensive battery of paper-and-pencil tests and computerized tasks tapping spatial exploration. Two test sessions were for baseline purposes, after which a placebo (two measurements) or guanfacine (two measurements) was given. Two out of the three patients showed clear improvements in both tasks after the administration of guanfacine, but not after the placebo intake. Both patients also showed an improved ability to sustain attention during visual exploration following guanfacine. The authors attribute the absence of benefit for the third patient to the dorsolateral-prefrontal localization of his lesion, because animal research has evidenced that guanfacine exerts its beneficial effect through this area of the brain.

## Prism Adaptation

In the past decade, various authors investigated the effects of PA (a.o. Frassinetti et al., 2002; Serino et al., 2007, 2009; Vangkilde and Habekost, 2010 – see Table S1 in Supplementary Material) in neglect, as introduced in a seminal study by Rossetti et al. (1998). In PA, mostly rightward displacing prism goggles are used. Patients are asked to point to targets that are placed in front of them. The leftward compensatory shift in straight ahead pointing that is observed after removal of the prism goggles (i.e., the negative aftereffect) has been reported to alleviate neglect symptoms on paper-and-pencil tasks for some minutes after one training session (Rossetti et al., 1998), although Rousseaux et al. (2006) found no specific effects in a similar one-session study. PA is thought to create plastic changes in the sensori-motor system (Luauté et al., 2006b) and realignment of the egocentric coordinate system (Redding and Wallace, 2006) by means of the spatially remapping of patients’ repeated pointing movements toward targets while they wear prism glasses, shifting the field of view to the right. Thus, PA may reduce the ipsilesional rightward bias that characterizes RH neglect (Rode et al., 2003). For instance, in some uncontrolled trials, changes have been reported in eye movements (Shiraishi et al., 2008, 2010), global versus local processing of space (Bultitude et al., 2009) and wheelchair navigating toward left targets (Watanabe and Amimoto, 2010). However, a clear and unambiguous explanation of the working mechanism of PA is still lacking (Newport and Schenk, 2012).

Various authors investigated whether short-term ameliorations after PA could be converted into long-term therapeutic improvement. For instance, in a study by Frassinetti et al. (2002), seven neglect patients performed a pointing task wearing prismatic lenses in twice-daily sessions over a period of 2 weeks. Improvements on a series of paper-and-pencil and behavioral tests were observed in these patients, but not in six untreated controls. Training effects in the PA group were maintained till a final measurement 5 weeks after treatment, except in one patient who did not show the adaptation effect and had an unstable aftereffect. On the other hand, in a randomized trial, Nys et al. (2008) found greater improvement on paper-and-pencil tasks in acute neglect patients receiving PA for 4 days in a row when compared to control patients who did not, but this difference had disappeared after 1 month.

Using protocols of 2 weeks of repeated training sessions, longer lasting effects have been observed in other studies. For instance, Serino et al. (2009) compared PA to a neutral pointing control training in two matched groups of neglect patients. After 2 weeks of neutral pointing, the control group also received PA training. It was observed that patients’ performances on paper-and-pencil tasks improved after both PA and neutral pointing, but the improvement was significantly more pronounced after PA. Moreover, after a second period of training using PA, the control group further improved up to the level reached by patients in the PA group. Improved performances on paper-and-pencil tasks were still observed a month after PA training.

Mizuno et al. (2011) conducted a RCT, comparing an experimental group (*N* = 20) of subacute neglect patients receiving PA training twice daily for 2 weeks to a control group (*N* = 18) that received similar training with neutral glasses. Pre- and post-training measures included the BIT, CBS, and FIM. Significantly more improvements on the FIM were observed in the PA group and significantly more improvement of both BIT and FIM in a subgroup with mild neglect symptoms receiving PA training. Effects lasting up to rehabilitation discharge (ranging from several weeks till few months after training) were observed.

However, in a similar RCT, Turton et al. (2010) found no differences between 16 post-acute neglect patients receiving a 2-week PA training and 18 patients receiving placebo treatment (i.e., wearing flat plain glasses) on neither self-care nor BIT performance, although both groups performed better after training than before.

In a study performed by Fortis et al. (2010), a comparison was made between a control condition consisting of a classic adaptation method (i.e., repeated pointing; Frassinetti et al., 2002) and an experimental adaptation method, involving ecological visuomotor activities. These were tasks like collecting coins, assembling puzzles, threading a necklace, and serving a cup of tea. Ten RH neglect patients were alternately assigned either to a program of 1 week of experimental followed by 1 week of control training or vice versa. Assessment tasks were administered at 1 week before treatment, at the beginning and ending of each treatment week and 1, 2, and 3 months after the end of treatment. Patients in both groups showed equal improvements after training on various neglect measures, the CBS and FIM. No relationship was found between neglect recovery and duration and disease.

Finally, PA has also been investigated in addition to other treatment methods, for instance neck muscle vibration. Saevarsson et al. (2010) applied neck muscle vibration in two groups of six RH neglect patients that were semi-randomly assigned to one of two conditions. Patients in both conditions received neck muscle vibration during a 20-min session. The experimental group received neck muscle vibration combined with PA for the same amount of time. Patients in both groups showed improved performance on a visual search task after treatment, but the patients that underwent the combined intervention showed clear improvements on visual search paper-and-pencil neglect tests that were not present in the group that only received neck vibration.

Various reviews on PA as a treatment method for neglect have been published recently (specifically Barrett et al., 2012; Newport and Schenk, 2012; Jacquin-Courtois et al., 2013). In each of these reviews, it is concluded that PA might be an effective therapy for patients with neglect. However, Barrett et al. (2012) emphasize that PA is not yet ready for broad administration in stroke rehabilitation and that it might be applied specifically for subgroups of patients presenting with motor-intentional “aiming” deficits. Newport and Schenk (2012) conclude that PA is only effective if training consists of 10 or more PA sessions. They argue that PA thus has become more and more similar to other, more traditional forms of neglect rehabilitation and might not fulfill initial promises. The authors stress the need for more research into the working mechanism of PA as well as the direct comparison with other rehabilitation techniques and more thorough investigation of ecologically relevant and long-term effects (see Shiraishi et al., 2010 for an exception: these authors performed a long-time follow-up using ecological measures). Fortis et al. (2010), based on the lack of a relationship between improvements after PA and duration of disease in their study, suggest that the treatment should be started as soon as clinically feasible and that the issue of post stroke intervals should be further explored. Finally, Jacquin-Courtois et al. (2013), despite some warnings about an ideal regime remaining to be defined more exactly, provide some practical guidelines for prism use in clinical practice. For instance, they recommend that 10–20 training sessions consisting of at least 60 pointing movements using sufficiently strong goggles (inducing at least 10° of visual displacement; see also Mancuso et al., 2012) are applied and that training only be given to patients showing a sufficient amount of aftereffect. Also, they indicate that the combination of techniques might provide future challenges as well as promises in neglect rehabilitation.

## Virtual Reality

Virtual reality has been defined as “an advanced form of human-computer interface that allows the user to ‘interact with’ and become ‘immersed in’ a computer-generated environment in a naturalistic fashion” (Laver et al., 2011). In stroke rehabilitation, VR techniques have been evaluated predominantly in studies designed to improve motor function rather than cognitive function or activity performance. For instance, in their recent Cochrane review on the use of VR in rehabilitation, Laver et al. (2011) found limited evidence that the use of VR and interactive video gaming may be beneficial in improving arm function and ADL function when compared with the same dose of conventional therapy. They indicate that it is unclear at present which characteristics of VR are most important and that it is unknown whether effects can be sustained in the longer term.

In neglect patients, VR has been recently applied both for diagnostic purposes (Broeren et al., 2007; Buxbaum et al., 2008, 2012; Jannink et al., 2009; Kim et al., 2010; Van Kessel et al., 2010, 2013a; Fordell et al., 2011; Peskine et al., 2011; Dvorkin et al., 2012) and as a rehabilitation tool (Webster et al., 2001; Castiello et al., 2004; Katz et al., 2005; Ansuini et al., 2006; Kim et al., 2007, 2011; Smith et al., 2007; Sedda et al., 2012; Van Kessel et al., 2013b). In their review on the use of VR in the assessment and treatment of neglect, Tsirlin et al. (2009) argue that an important benefit of VR technologies is that they provide rich and realistic environments with a high level of control over their parameters and thus allow for training in a safe and cost effective way.

As a rehabilitation tool in neglect, VR has for instance been used to simulate grasping in space using a hand-motion tracking device (Castiello et al., 2004; Ansuini et al., 2006). In the VR tasks, dissociations were induced between real and simulated locations of stimuli, thus distorting the patients’ representation of space. The authors argue that this might lead to the formation of novel neural circuitry governing visuo-proprioceptive integration, bearing resemblance to the effects of PA. Also Sedda et al. (2012), in a case study training a patient using a VR searching and grasping task, suggest that specific cognitive rehabilitation using VR may favor plastic reorganization of the brain.

In four case studies, Smith et al. (2007) had patients with mild neglect play computer games using a device translating the subjects’ movements into the movements of an avatar on the screen. They report small improvements on paper-and-pencil tasks after six weekly training sessions. More recently, Kim et al. (2011) trained 24 RH neglect patients, randomly assigned to either a VR group or a control group. The VR group received training involving playing interactive computer games, the control group received conventional neglect therapy (i.e., reading, drawing, making puzzles). Both groups received therapy for 30 min a day, 5 days a week for 3 weeks. Differences in test scores between the start and end of training were significantly higher in the experimental group for two out of four measures (paper-and-pencil tasks and rating scales) that were used. The authors suggest that VR training may have a beneficial effect on unilateral spatial neglect after stroke.

Virtual reality has been applied to train patients to voluntarily compensate for their disorder in specific daily life situations. For instance, better performance on a real-life wheelchair obstacle course and less falling and accidents were reported in 20 neglect patients who received training by means of a desktop computer program involving sustained attention tasks and simulated wheelchair obstacle courses, compared to 20 untrained control patients with neglect (Webster et al., 2001). Katz et al. (2005) used a 12 session computer desktop-based training in which patients were required to press a button the moment they thought it safe to cross a virtual street. A group of 11 trained subjects improved more than eight controls on the practiced task and looked to the left more often in real street crossing after training, whilst performances on paper-and-pencil tasks did not differ between groups. In a preliminary study using a head-mounted device simulating crossing a street, Kim et al. (2007) found more symmetrical performance on the practiced task in 10 neglect patients after an unspecified number of training sessions, lasting till 3-month follow-up. Sedda et al. (2012), in a case study training a patient using a VR searching and grasping task, found significant amelioration on neuropsychological tests and self-reports of daily functioning. The authors suggest that specific cognitive rehabilitation using VR may favor plastic reorganization of the brain.

On the other hand, Akinwuntan et al. (2010) observed no differences between two groups of stroke patients with and without neglect participating in a large RCT (*N* = 69), receiving either simulator-based driving-related training or non-computer-based cognitive training for 15 h over 5 weeks. In fact, both groups showed significant but similar improvement in performance on a test of driving-related visual attention skills after training and benefits lasted up to 6 months after stroke. Van Kessel et al. (2013b) conducted a study in which visual scanning training (based on Pizzamiglio et al., 1990, 1992) was compared to an experimental condition consisting of a combination of visual scanning training and a VR driving simulator task. Twenty-nine subacute right hemisphere stroke patients were semi-randomly assigned to one of both conditions. On various neglect and driving simulator tasks, significant improvements after training were observed in both groups taken together, but no differences between groups were found. Thus, despite some promising results, no convincing evidence for the effectiveness of VR training has been reported till now.

## Conclusion

The last decade has seen the emergence of four new treatment approaches in neglect rehabilitation: NIBS, pharmacological therapies, PA, and VR training have made their way through older and well-established treatment methods like visual scanning training and limb activation training. In the present review, a broad overview is given of the studies undertaken since the last decade to evaluate the effectivity of these new approaches in visuospatial neglect rehabilitation. A limitation of this survey is its non-systematic character, insofar as we did not include a scoring of the levels of evidence based on the used methodology. Therefore, it may contain a selection bias. Also, no meta-analyses of aggregated data are presented. Still, we believe that some conclusions may be drawn from the reported studies.

In general, the benefits of the new neglect rehabilitation techniques seem to be significant and may last for variable periods of time. In some cases the effects are still present after 2 months, especially when multiple training sessions have been applied. Unfortunately, in the majority of studies no long-term measurements have been performed. Moreover, visuospatial neglect is not an isolated symptom, but is often associated with symptoms like anosognosia, hemiparesis, or somatoparaphrenia. The absence of evaluation of these symptoms is clearly a limitation of the studies reviewed in the present paper. And lastly, the small sample sizes, the regular absence of control conditions and the explorative character of several studies restrict the reliability of their conclusions. So, despite encouraging results yielded by these new approaches Kerkhoff and Schenk’s (2012) statement that “the initial hope for a quick cure for neglect after only one or a handful of treatment sessions has turned out to be unrealistic” still sounds true.

We think that the studies that we have reviewed are often proof-of-principle studies into new approaches in neglect rehabilitation. Therefore, much more research is needed in which several issues will have to be taken into account.

First, there is the point of generalization in time. Most studies have shown positive effects, but only for a limited time-window. In future studies it would be desirable to extend effect measurements up to 6 months after treatment, in order to establish the longer-term effects of the different treatments. TBS seems a promising candidate for LTP or depotentiation of synaptic plastic changes in patients with visuospatial neglect. More in general, one of the problems with novel treatments is also that they could be diversely effective depending on the time of treatment. Most studies do not consider this variable. A hypothesis might be that treatments stimulating an active participation by the patient might favor brain plasticity, but only in the chronic stage of the illness. Therefore, bottom-up techniques like drug treatments, PA, and NIBS (when no active tasks are used) might be more fitting in the acute stage, whereas VR treatments requiring an active (top-down) participation could be more useful in the chronic stage.

Second, there is the issue of measurement instruments. In the majority of studies, therapy effects are measured with neuropsychological tests. Only exceptionally, the efficacy of a treatment is also assesses on daily life neglect behavior. A more frequent use of instruments like the Catherine Bergego Scale (Azouvi et al., 2003) or the functional evaluation of neglect with a Semistructured Scale (Zoccolotti and Judica, 1991) is needed to evaluate the impact of treatment on the daily life neglect behavior of patients. This also applies to the above mentioned issue of subtle neglect revealed by increasing attentional load. Most studies use tests (e.g., paper-and-pencil) that are too coarse to identify these subtle forms of neglect and so these patients are not included in trials of neglect rehabilitation.

Third, true replication studies are needed. Within the approaches that we have reviewed, the difficulty was to make a true comparison between studies, due to differences in methodology, design, and patient populations. Although replication studies may seem less appealing, they are sorely needed in a field were much things are novel and risk to remain novel. Also, the number of studies that directly compare the effects of different training methods is very limited. Recently, Priftis et al. (2013) made an attempt to compare visual scanning training, limb activation training, and PA. Thirty-three neglect patients were quasi-randomly assigned one of these three training methods. All patients received 20 training sessions (two daily sessions during 2 weeks). Improvements on tests assessing the peripersonal space in everyday life activities were observed over the three conditions. However, no different treatment effects were observed between groups. Thus, the authors suggest that all three treatments might be considered as valid rehabilitation methods for neglect. We recommend that more studies investigating the differential effects of various training techniques are conducted.

Finally, Kerkhoff and Schenk’s (2012) suggestion, that the true challenge will be to find the best combination of treatments for a given patient in order to maximize benefits, has not lost its strength. Likewise, Saevarsson et al. (2011) argue that combining various therapeutic techniques might be worthwhile, because of the heterogeneity of the neglect syndrome. A good mixture of treatment ingredients would be largely facilitated by more fundamental knowledge about the mechanisms of visuospatial neglect and research into these mechanisms should continue with the same intensity in the future. This knowledge might facilitate the choice of treatments suitable for individual patients.

## Conflict of Interest Statement

The authors declare that the research was conducted in the absence of any commercial or financial relationships that could be construed as a potential conflict of interest.

## Supplementary Material

The Supplementary Material for this article can be found online at http://www.frontiersin.org/journal/10.3389/fnhum.2013.00780/abstract
