# Supplementary material for: Novel Insights in the Rehabilitation of Neglect
Source: Front Hum Neurosci. 2013 Nov 15;7:780. doi: 10.3389/fnhum.2013.00780 (PMC3828556; doi:10.3389/fnhum.2013.00780)
Supplement: Supplementary file 1 [file 52070_Fasotti_DataSheet1.PDF]

Table S1: Summary of neglect rehabilitation studies on non-invasive brain stimulation, prism adaptation and virtual reality currently available

NIBS

	Design	Patients	Post onset	Control condition	Experimental condition	Outcome measures	Measurements	Results	Long-term results
Oliveri et al., 2001	Within subjects design	N=7, 5 RBD (right brain damage) and 2 LBD (left brain damage) patients with visual neglect	1-24 weeks	Sham rTMS	rTMS parietal cortex of the unaffected hemisphere, trains of 10 pulses, 25 Hz, duration 400 ms	Judgment of prebisected lines	Online	Transient decrease of the magnitude of neglect, as expressed in the number of judgment errors.	
Brighina et al., 2003	Within subjects design	N= 3, 3 RBD (right brain damage) patients with visual neglect, 2 with temporo-parietal lesions, 1 with a fronto-parietal lesion	3-5 months	No treatment period	rTMS left posterior parietal cortex, trains of 900 pulses, 1 Hz, 7 sessions over 2 weeks	Judgment of prebisected lines, clock drawing, line bisection	Time 1: 15 days before treatment, Time 2: start of treatment, Time 3: end of the treatment, Time 4: 15 days after treatment	Significant improvement of visuospatial performance (line judgment, clock drawing and line bisection)	Improvement still present after 15 days.
Shindo et al., 2006	Within subjects design	N=2, 2 RBD (right brain damage) patients with visual neglect, 1 fronto-parietal, 1 parieto-temporal	6 months		rTMS left posterior parietal cortex, trains of 900 pulses, 0.9 Hz, 6 sessions over 2 weeks	Behavioral Inattention Test (conventional and behavioral parts)	Time 1: 2 weeks before treatment, Time 2: day before 1 session, Time 3: day after first session, Time 4: 2 weeks after last session, Time 5: 4 weeks after last session, Time 6: 6 weeks after last session	Better scores on BIT tests (conventional and behavioral), not statistically tested	6 weeks after last rTMS session scores were still higher than at baseline (not statistically tested)
Koch et al., 2008	Control group design	E: N=10 RH neglect patients<br><br>C: N=5 RH non-neglect patients	1-6 months	Same stimulation as experimental group	rTMS posterior parietal cortex, trains of 600 pulses, 1 Hz, 1 session	Amplitude of Motor Evoked Potentials produced by a test pulse over left motor cortex (M1), naming of chimeric objects	MEPs before and 1 minute after stimulation, chimeric objects before and after stimulation	Significantly better naming of chimeric objects after stimulation in the neglect group, reduced hyperexcitability upon the left posterior parietal -M1 cortex	
Song et al.,2009	Control group design	E: N=7 RH stroke patients (of mixed etiology) with neglect<br><br>C: N=7 RH stroke patients (of mixed etiology) with neglect	15 days-2 months	Conventional rehablitation	Conventional rehabilitation + left posterior parietal cortex rTMS trains of 450 pulses, 0.5 Hz, 2 sessions per day for 2 weeks	Line bisection and line cancellation	Time 1: 2 weeks before treatment, Time 2: beginning of treatment, Time 3: end of treatment, Time 4: 2 weeks after treatment	Improvement of visual spatial neglect in the experimental group , not in the control group. Comparison of groups showed only post-treatment difference on the cancellation task.	Improvement still present after 2 weeks in experimental group.

	Design	Patients	Post onset	Control condition	Experimental condition	Outcome measures	Measurements	Results	Long-term results
Lim et al., 2010	Control group design	E: N=7 RH stroke patients (mixed etiology and localization) with neglect<br><br>C: N=7 RH stroke patients (mixed etiology and localization) with neglect	9-470 days	Behavioral therapy (standardized, e.g. visual scanning + feedback)	Behavioral therapy + rTMS trains of 900 pulses, 1 Hz, 5 days per week for 2 weeks	Line bisection test (Schenkenberg) and line cancellation (Albert test)	Time 1: 1 day before interventions<br><br>Time 2: 1 day after last rTMS session	Improvement in line bisection greater in experimental than control group. This was not the case for line cancellation.	
Ko et al., 2008	Within subjects design, double-blind, crossover, sham controlled	N=15 RH stroke patients (mixed etiology and localization) with neglect	29 to 99 days	1 session of sham stimulation of 2.0 mA for 10 seconds, then current turned off	1 session of right posterior parietal cortex anodal tDCS of 2.0 mA for 20 min	Line bisection, shape-unstructured cancellation test, letter-structured cancellation test	Before and immediately after tDCS and sham stimulation	Improvement in line bisection and shape-unstructured cancellation, not in letter-structured cancellation.	
Sparing et al., 2010	Within subjects design, order of stimulation counterbalanced across subjects	N=10 RH stroke patients (mixed etiology and localization) with neglect	2.9 to 6.4 months	Sham stimulation in the lesioned right hemisphere	1.tDCS anodal intact left hemisphere<br>2. tDCS cathodal intact left hemisphere<br>3. tDCS anodal lesioned right hemisphere<br>4. Sham lesioned right hemisphere (control condition)	TAP subtest neglect, line bisection	Directly before and after each stimulation session	Significant effects of tDCS cathodal in intact left hemisphere and anodal tDCS in lesioned right hemisphere on line bisection task performance, not on TAP neglect subtest	
Nyffeler et al., 2009	Mixed design (partially within subjects and partially between subjects)	N=11 RH neglect patient (mixed etiology and localization)	0.4 to 36.1 months	1. 2 sham TBS trains over intact left hemisphere<br><br>2. No intervention	1. 2 TBS trains of 801 pulses delivered in 267 bursts over intact left hemisphere. Each burst contained 3 pulses at 30 Hz, total duration 44s<br><br>2. 4 TBS trains	Subtask of the Vienna Test System, measures peripheral visual attention during simultaneous central tracking	Directly before, 1 hour after, 8 hours after (2 TBS trains), 32 hours after (4 TBS trains), 96 hours after (4 TBS trains)	2 TBS trains increased nr. of perceived left visual targets and decreased reaction time significantly after 1 hour<br><br>4 TBS trains had the same effect	Both effects of 2 TBS trains lasted for 8 hours<br><br>Both effects of 4 TBS trains lasted for 32 hours, but had disappeared after 96 hours

	Design	Patients	Post onset	Control condition	Experimental condition	Outcome measures	Measurements	Results	Long-term results
Koch et al., 2012	Control group design: randomized, double-blind, sham-controlled study.	E: N= 9 RH ischemic stroke patients with neglect<br><br>C: N= 9 RH ischemic stroke patients with neglect	Both groups "subacute"	Conventianla neglect therapy based on computerized visual scanning training + sham cTBS on left posterior parietal cortex	Conventional neglect training + cTBS on left posterior parietal cortex consisting of 3 pulse bursts at 50 Hz every 200 msec for 40s, twice daily for 10 days (two weeks)	1.Behavioral Inattention Test total score (conventional + behavioral)<br><br>2. MEP to assess connections between left PPC and primary motor cortex (M1)	Time 1: 1 hour before first session of stimulation<br><br>Time 2: Monday following 2 weeks of stimulation<br><br>Time 3: 4 weeks after beginning of stimulation	1.Experimental group: improved neglect scores by 16,3 %, sham control group not.<br><br>2. Excitability of PPC-M1 connection reduced following real but not sham cTBS	1.Improvement of 22.6% in neglect scores after 4 weeks in experimental group, no improvement in sham control group.<br><br>2. Excitability of PPC=M1 connection stil reduced in real cTBS group, not in sham group
Cazzoli et al., 2012	Control group design: randomized, double-blind, sham-controlled study.	E1: N=8 RH stroke patients with neglect (ischemic and haemorrhagic stroke)<br><br>E2: : N=8 RH stroke patients with neglect (ischemic and haemorrhagic stroke)<br><br>C: : N=8 RH stroke patients with neglect (ischemic and haemorrhagic stroke)	Mean 26.63 days (SE of mean 4.44 days)	No treatment group, only conventional visuospatial exploration training 1 hour per day	E1:Conventional spatial exploration training 1 hour per day, cTBS then sham<br><br>E2:conventional spatial exploration training 1 hour per day, sham then cTBS<br><br>(TBS consisted of trains of 801 pulses delivered in 267 bursts over intact left hemisphere. Each burst contained<br><br>3 pulses at 30 Hz, total duration 44s. In total 8 trains of cTBS were applied over 2 days)	1)Subtask of the Vienna Test System, measures peripheral visual attention during simultaneous central tracking<br><br>2.Catherine Bergego Scale<br><br>(neglect in ADL-activities)<br><br>3. Several paper- and –pencil neglect tasks	Time 1: baseline<br><br>Time 2: after cTBS (group E1), after sham (group E2)<br><br>Time 3: after sham (group E1), after cTBS (group E2)<br><br>Time 4: one week after T3<br><br>Time 5: two weeks after T3 (only group E2)	1.In both experimental groups neglect on the Subtask of the Vienna Test System was significantly reduced after cTBS, not after sham stimulation. In the no- treatment group no reduction of neglect was assessed.<br><br>2.In both experimental groups neglect on the Catherine Bergego Scale was significantly reduced after cTBS, not after sham stimulation. Mean improvement was 37%. In the no- treatment group no reduction of neglect was assessed<br><br>3.In paper-and-pencil tests the same trend was visible: improvements after cTBS, not post sham. No-treatment produced no reduction of neglect.	1. Omitted targets in Subtask of Vienna Test system still significantly reduced at Time 4 in both E1 and E2 groups and at Time 5 (group E2 only)<br><br>2. Reduction of neglect in Catherine Bergego Scale still present at Time 4 in both E1 and E2 groups at Time 4 and 5 (group E2 only)<br><br>3. Same results as 1 and 2 for paper-and-pencil tests

PA

	Design	Patients	Post onset	Control condition	Experimental condition	Outcome measures	Measurements	Results	Long-term results
Rossetti et al., 1998	Control group design	E: N=6 RH neglect patients<br><br>C: N=6 RH neglect patients	3 weeks-14 months	one pointing session wearing flat glasses	one pointing session wearing prismatic glasses	line bisection, line cancellation, copying, drawing, reading	directly before and after the pointing sessions and after two hours	Significantly better performance directly after training and after two hours in the E but not the C group	
Frassinetti et al., 2002	C patients were recruited from one hospital, E patients from another	C: N=6 neglect patients<br><br>E: N=7 neglect patients	3-27 months	no treatment	20 min pointing task wearing prismatic lenses in twice-daily sessions over a period of 2 weeks (a total of 20 sessions)	cancellation tests, reading, Fluff test, room description, objects reaching, Motricity Index	before treatment, 2 days, one week and five weeks after treatment	improvements in the E, but not the C condition on paper-and-pencil tasks, room description and objects reaching, except in one E patient who did not show post-adaptation after-effect	training effects in the E group were maintained five weeks after treatment
McIntosh et al., 2002	single case study	N=1 RH neglect patient	9 months		three weekly pointing sessions wearing prismatic lenses	cancellation, copying, drawing and bisection, haptic circle centring task	Pre-test, 2 hours after first PA, immediately after PA session 2, 2 hours after PA session 3	improvements were obtained on most tests except reading	
Dijkerman et al., 2004	single case study	N=1 RH neglect patient	3 months		two single pointing sessions wearing prismatic lenses<br><br>(3 weeks between sessions)	BIT subtests, pressure sensitivity, passive finger position sense	3x baseline (once a week); 30 minutes, 1 and 3 weeks after first PA; 30 minutes after second PA	improvements in pressure sensitivity and proprioception were observed directly after PA.	Significantly improved performance was found for position sense three weeks after PA
Angeli, 2004	Control group design	E: N=8 RH neglect patients<br><br>C: N=5 RH neglect patients		one pointing session wearing flat glasses	one pointing session wearing prismatic glasses	eye movements in reading tasks	directly before and after the PA session	Improvements of neglect dyslexia, left-sided exploration and amplitude of the first left-sided saccade in the E but not the C group.	

	Design	Patients	Post onset	Control condition	Experimental condition	Outcome measures	Measurements	Results	Long-term results
Rousseaux et al., 2006	within subjects design	N=10 neglect patients,<br><br>N=8 matched healthy controls	17 to 102 days		2 pointing sessions (1 week in between) with neutral and prismatic lenses in a randomized order	reading, bells test, line bisection, drawing	baseline, 1 hour pre-PA; 5 minutes, 3 hours, 1 day and 3 days after PA	Errors were more frequent and an improvement was found in the late sessions, but without any specific effect of wearing prisms.	
Serino et al., 2007	control group design; pts divided according to the level of after-effect during one week of training	N= 21<br>neglect patients	3-96 months		20 min pointing task wearing prismatic lenses in daily sessions over a period of 2 weeks (a total of 10 sessions)	cancellation tests, reading, Fluff test, room description, objects reaching, Motricity Index, tactile extinction test,proprioceptive scale	before treatment, 1 week, 1 month, 3 months and 6 months after treatment	amelioration on conventional and behavioural tasks and for neglect dyslexia, exploration of personal space, oculomotor responses and tactile attention but not proprioceptive sensitivity and motor functions. Patients showing poor prism adaptation effects during the first week of PA also showed less amelioration of neglect.	Improved performances were generally observed till 6 months after training
Shiraishi et al., 2008	within subjects design	N=7 neglect patients	12 - 84 months		8-week (total: mean 28 hours) intervention using prismatic glasses, performing various activities (ring tossing, pegboard, ball throwing, darts)	eye movements, bias in center of gravity in the standing position, regional cerebral blood flow	before, immediately after, 1 day and 4 weeks after treatment	more eye movements on the neglected side, center of gravity moved to the left and forward, rCBF increase	effects up to 6 weeks after treatment
Shiraishi et al., 2010	follow-up assessment for Shiraishi et al., 2008	N=5 neglect patients	14 - 84 months		see above	see above, +: cancellation tasks; Barthel Index; Lawton's IADL scale; ADL interview	2-3,5 years after treatment		eye movement and center of gravity were stable; improvement in cancellation and line bisection tests. All subjects showed improvement in ADL. Two subjects could return to work.

	Design	Patients	Post onset	Control condition	Experimental condition	Outcome measures	Measurements	Results	Long-term results
Nys et al., 2008	RCT	C: N=6 neglect patients<br><br>E: N=10 neglect patients	2-23 days	placebo for four days in a row	PA for four days in a row	line bisection, letter cancellation, scene copying; after one month: BIT and Barthel Index	immediately before and after each treatment session and after 1 month	E patients improved faster on spatial tasks	No differences between groups at one month post-treatment.
Jacquin-Courtois et al., 2008	single case study	N=1 RH neglect patient	> 3 months		one PA session	pointing straight-ahead, line cancellation test,line bisection task and wheel-chair driving	two times 3 days and one time just before intervention; directly after prism removal and 1h, 24h, 48h, 72h, 96h later	improvement of wheel-chair driving aswell as of classical tests, partly diminishing after 48-96 hours	
Serino et al., 2009	pseudorandomly, matched groups; C group also received E training after two weeks of C training	C: N=10 neglect patients<br><br>E: N=10 neglect patients	1-60 months	20 min pointing task wearing neutral lenses (10 sessions)	20 min pointing task wearing prismatic lenses in daily sessions over a period of 2 weeks (a total of 10 sessions)	BIT, Bells test, reading	before treatment, 1 week after each treatment and 1 month after E treatment	performances on paper-and-pencil tasks improved after both E and C training, but improvement was significantly more pronounced after E treatment. After E training, the C group further improved up to the level reached by the E group.	Improved performances on paper-and-pencil tasks were still observed a month after PA training.
Vangkilde et al., 2010	control group design; pseudo-randomly	E: N=6 RH neglect patients<br><br>C: N=5 RH neglect patients	6-138 months	8 hours of general cognitive rehabilitation	15 min pointing task wearing prismatic lenses in twice-daily sessions over a period of 2 weeks (a total of 20 sessions)	line bisection, star and letter cancellation, Baking<br><br>Tray, copying figures, Cupboard test, subjective questionnaire	baseline, shortly after training, and five weeks later	Improvements in the E compared to the C group were observed on three out of five paper-and-pencil tests, the cupboard test and the subjective questionnaire	Ameliorated results remained stable till 5 weeks after training
Bultitude et al., 2010	case study, multiple baseline	N=1 patient with LH neglect<br><br>N=8 healthy matched controls	3 months	three testing sessions on one day: no treatment, sham treatment, PA	eight testing sessions; sham treatment (day 2) and prism treatment (day 7)	day 1 , 2, 2, 6, 7, 7, 8, 18	line bisection	PA aftereffects in all participants; 5% improvement on the bisection task in the patient with a tendency to return to baseline 11 days later	

	Design	Patients	Post onset	Control condition	Experimental condition	Outcome measures	Measurements	Results	Long-term results
Watanabe & Animoto, 2010	prospec-tive cohort study	N=10 neglect patients	5-38 days		one PA session	directly before and after PA	wheelchair navigating toward 1) the middle, between to targets 2) targets on different horizontal positions	improvement on both tasks after PA	
Turton et al., 2010	control group design	C: N=18 neglect patients<br><br>E: N=16 neglect patients	mean 46 days	daily pointing sessions for two weeks (10 sessions), neutral glasses	daily pointing sessions for two weeks (10 sessions) wearing prism glasses	four days and eight weeks after treatment	Behavioral Inattention Test, Catherine Bergego Scale	no differences between groups, although both groups performed better after training than before	no differences between groups
Fortis et al., 2010	cross-over; both control and experimen tal training in random order	N=10 RH neglect patients	1-10 months	repeated pointing wearing prisms	ecological visuomotor activitieswearing prisms: collecting coins, assembling puzzles, threading a necklace and serving tea	1 week before treatment, beginning and ending of each treatment week; 1, 2, 3 months after treatment	cancellation, reading, and drawing tasks, CBS and FIM	Patients in both groups showed equal improvements after training	Recovery took place after the first week, continued in the second week, and was stable at the follow-up of 3 months
Saevarsson et al., 2010	semi-randomly	C: N=6 neglect patients<br><br>E: N=6 neglect patients	3-57 months	neck muscle vibration during twenty minutes	neck muscle vibration combined with PA during twenty minutes	before and after treatment	computerized visual search task, standard neglect tests	both groups showed improved visual search, but E patients showed also clear improvements on paper and pencil neglect tests	
Mizuno et al., 2011	multicente r, double-masked RCT	E: N=20 neglect patients<br><br>C: N=18 neglect patients	mean 65 days	repetitive pointing twice daily for two weeks (20 sessions), neutral glasses	repetitive pointing twice daily for two weeks (20 sessions) wearing prism glasses	study entry, just after intervention<br><br>and at discharge	Behavioral Inattention Test, Catherine Bergego Scale, Functional Independence Measure	more improvements on the FIM were observed in the E group and more improvement of both BIT and FIM in a E subgroup with mild neglect	Effects lasting up to rehabilitation discharge (several weeks till few months after training)
Fortis et al., 2011	within subjects design	N=5 RH neglect patients	2-5 weeks		two prism adaptation sessions on consecutive days	line bisection via video screen; 'where' vs. 'aiming' conditions created by mirroring	before and after the two days of prism adaptation	significant improvement in 'aiming' spatial bias, with no effect on 'where' spatial bias	

	Design	Patients	Post onset	Control condition	Experimental condition	Outcome measures	Measurements	Results	Long-term results
Nijboer et al., 2011	single case study	N=1 RH neglect patient	6 years		daily PA during three monts	perimetry, BIT line bisection and star cancellation,	start of prism adaptation; immediately, 3, 6 and 24 months after PA	improvements on al measurements were observed after PA	
Mancuso et al., 2012	RCT	N=29 RH neglect patients	acute	pointing exercises wearing neutral lenses for 5 sessions of 30 minutes during one week	pointing exercises wearing 5˚ prismatic lenses for 5 sessions of 30 minutes during one week	visuospatial tests	before and after intervention	improvement within the two groups; however, the prismatic lenses of only five degrees did not contribute to the variation in performance	
Luauté et al., 2012	within subjects design; single session	N=5 RH neglect patients<br>N= 6 healthy controls	1 -2.5 months		single session of exposing simple manual pointing to left-deviating prisms	line cancellation, balloon test, line bisection, copy a scene, drawing, reading	before prism adaptation; immediately and 2 hours after	contrary to healthy controls, none of the patients showed a reliable change in straight ahead pointing, nor performance of paper-and-pencil tasks immediately or 2 hours after prism adaptation	

VR

	Design	Patients	Post onset	Control condition	Experimental condition	Outcome measures	Measurements	Results	Long-term results
Webster et al., 2001	case-control study	E: N=20 RH neglect patients<br>C: N=20 RH neglect patients	mean 166 days	no training	12 to 20 sessions of about 45 minutes; computerized detection tasks, simulated wheelchair obstacle courses	computerized tasks, real-life wheelchair obstacle course, occurrence of falls and accidents	before and after training (unspecified)	better performance on a real-life wheelchair obstacle course and less falling and accidents were reported in the E group compared to the C group after training	
Castiello et al., 2004	within subjects design	N=6 RH neglect patients<br>N=6 healthy controls	56-66 days		3 sessions on 1 day; reaching and grasping wearing a motion-tracking glove, while observing a virtual hand grasping a virtual object	accuracy on the trained task	first and third session	neglect patients showed a significant increase in the percentage of correct left responses after the left-incongruous trials	

	Design	Patients	Post onset	Control condition	Experimental condition	Outcome measures	Measurements	Results	Long-term results
Katz et al., 2005	RCT	E: N=11 RH neglect patients<br><br>C: N=8 RH neglect patients	6-8 weeks	12 sessions of computerized visual scanning training during 4 weeks	twelve sessions of VR computer desktop-based training during four weeks; patients pressed a button when they thought it safe to cross a virtual street	practiced task, real life street crossing, paper-and-pencil tasks	before and after training (unspecified	the E group improved more than the C group on the practiced task and looked to the left more often in real street crossing after training, performances on paper-and-pencil tasks did not differ between groups	
Ansuini et al., 2006	within subjects design	N=6 RH neglect patients (3 dorsal fronto-parietal, 3 ventral temporo-parietal)<br><br>N=3 healthy controls	56-64 days		three sessions on one day; reaching to grasp either a real or a virtual object, wearing a motion-tracking glove	accuracy on the trained task	first and third session	only the FP group showed significant improvement in responses to leftward targets after left-incongruous trials	
Smith et al., 2007	series of case studies, double baseline	N=4 neglect patients (neglect not visible on BIT nor bells test)	13 months – 11 years		six weekly training sessions of computer games (detection tasks), user's image projected onto the screen	BIT, bells test; interview regarding the intervention	one week and directly before treatment; one week after treatment	small improvements on paper-and-pencil tasks (no statistical tests)	
Kim et al., 2007	within subjects design	N= 10 RH neglect patients<br><br>N=40 healthy controls	not specified		unspecified number of training sessions; head-mounted device projecting a detection task simulating crossing a street	practiced task	before and after training (unspecified)	more symmetrical performance on the practiced task (no statistical tests)	
Akinwuntan et al., 2010	RCT	E: N=33 RH neglect patients<br><br>C: N=36 RH neglect patients	mean 54 days	15 hours of non-computer-based cognitive training over 5 weeks	15 hours of simulator-based driving-related training over 5 weeks	Useful Field of View test	before and after training (unspecified), 3 months after training	both groups showed significant but similar improvement in performance on a test of driving-related visual attention skills after training	

	Design	Patients	Post onset	Control condition	Experimental condition	Outcome measures	Measurements	Results	Long-term results
Kim et al., 2011	RCT	E: N=12 RH neglect patients<br><br>C: N=12 RH neglect patients	7-44 days	30 minutes a day, 5 days a week for 3 wks; conventional neglect therapy (i.e. reading, drawing, puzzles)	30 minutes a day, 5 days a week for 3 wks; interactive computer games (detection tasks), user's image projected onto the screen	star cancellation, line bisection, CBS, Barthel index	24 hours before and after the three-week treatment	changes in star cancellation CBS in the E group were signifi cantly higher than those of the C group after treatment	
Sedda et al., 2012	single case study	N=1 RH neglect patient	1 year		Training for 4 weeks, one hour each day; patients' silhouet is pasted into various VR environments, thus creating searching and grasping tasks	attentional matrices, MMSE, line bisection, line cancellation	before treatment; immediately and 4 months after treatment	significant improvement on matrices and MMSE; partial improvement on line bisection, line cancellation	
Van Kessel et al., 2013	control group design, semi-randomized	E: N=14 RH neglect patients<br><br>C: N=15 RH neglect patients	mean 149 days	visual scanning training	combination of visual scanning training and a VR driving simulator task, thus creating a dual task	paper-and-pencil tests, semi-structured scale, subjective questionnaire, driving simulator tasks	one or two weeks before and after treatment	Patients in both groups taken together showed significant improvements on almost all tasks. However, no significant differences between groups were observed, neither before nor after training	
